# Differentiation of tumor versus peritumoral cortex in gliomas by intraoperative electrocorticography

**DOI:** 10.1093/neuonc/noaf082

**Published:** 2025-04-24

**Authors:** Belén Díaz-Fernández, David Henao-Herreno, Juan Nieto, Alesya Evstratova, Silvia Cases-Cunillera, Louise Deboeuf, Alexandre Roux, Edouard Dezamis, Marc Zanello, Bertrand Mathon, Carine Karachi, Alexandre Carpentier, Pascale Varlet, Johan Pallud, Laurent Capelle, Catalina Alvarado-Rojas, Michel Le Van Quyen, Gilles Huberfeld

**Affiliations:** Department of Neurology, Pitié-Salpêtrière Hospital, Paris, France; Panaxium SAS, Aix-en-Provence, France; Laboratoire d’Imagerie Biomedicale, Neuronal Connecivity and Plasticity, INSERM U1146, Paris, France; Institute of Psychiatry and Neuroscience of Paris (IPNP), INSERM U1266, Neuronal Signaling in Epilepsy and Glioma, Université Paris Cité, Paris, France; Center for Interdisciplinary Research in Biology, Collège de France, Paris, France; Institute of Psychiatry and Neuroscience of Paris (IPNP), INSERM U1266, Neuronal Signaling in Epilepsy and Glioma, Université Paris Cité, Paris, France; Center for Interdisciplinary Research in Biology, Collège de France, Paris, France; Institute of Psychiatry and Neuroscience of Paris (IPNP), INSERM U1266, Neuronal Signaling in Epilepsy and Glioma, Université Paris Cité, Paris, France; Center for Interdisciplinary Research in Biology, Collège de France, Paris, France; Institute of Psychiatry and Neuroscience of Paris (IPNP), INSERM U1266, Neuronal Signaling in Epilepsy and Glioma, Université Paris Cité, Paris, France; Center for Interdisciplinary Research in Biology, Collège de France, Paris, France; Institute of Psychiatry and Neuroscience of Paris (IPNP), INSERM U1266, Neuronal Signaling in Epilepsy and Glioma, Université Paris Cité, Paris, France; Institute of Psychiatry and Neuroscience of Paris, University Paris Cité, INSERM U1266, IMABRAIN, Paris, France; Department of Neurosurgery, GHU Paris Psychiatry and Neurosciences, Sainte-Anne Hospital, Paris, France; Laboratoire d’Imagerie Biomedicale, Neuronal Connecivity and Plasticity, INSERM U1146, Paris, France; Institute of Psychiatry and Neuroscience of Paris, University Paris Cité, INSERM U1266, IMABRAIN, Paris, France; Department of Neurosurgery, GHU Paris Psychiatry and Neurosciences, Sainte-Anne Hospital, Paris, France; Institute of Psychiatry and Neuroscience of Paris, University Paris Cité, INSERM U1266, IMABRAIN, Paris, France; Department of Neurosurgery, GHU Paris Psychiatry and Neurosciences, Sainte-Anne Hospital, Paris, France; Université Paris Cité, Paris, France; Institute of Psychiatry and Neuroscience of Paris, University Paris Cité, INSERM U1266, IMABRAIN, Paris, France; Department of Neurosurgery, GHU Paris Psychiatry and Neurosciences, Sainte-Anne Hospital, Paris, France; Department of Neurosurgery, Pitié-Salpêtrière Hospital, Paris, France; Department of Neurosurgery, Pitié-Salpêtrière Hospital, Paris, France; Department of Neurosurgery, Pitié-Salpêtrière Hospital, Paris, France; Department of Neuropathology, GHU Paris Psychiatry and Neurosciences, Sainte-Anne Hospital, Paris, France; Université Paris Cité, Paris, France; Institute of Psychiatry and Neuroscience of Paris, University Paris Cité, INSERM U1266, IMABRAIN, Paris, France; Department of Neurosurgery, GHU Paris Psychiatry and Neurosciences, Sainte-Anne Hospital, Paris, France; Department of Neurosurgery, Pitié-Salpêtrière Hospital, Paris, France; Department of Electronics Engineering, Pontificia Universidad Javeriana. Bogotá, Colombia; Laboratoire d’Imagerie Biomedicale, Neuronal Connecivity and Plasticity, INSERM U1146, Paris, France; Department of Neurology, Hopital Fondation Adolphe de Rothschild, Paris, France; Institute of Psychiatry and Neuroscience of Paris (IPNP), INSERM U1266, Neuronal Signaling in Epilepsy and Glioma, Université Paris Cité, Paris, France; Center for Interdisciplinary Research in Biology, Collège de France, Paris, France

**Keywords:** biomarker, electrocorticography, gliomas, power spectrum, tumors

## Abstract

**Background:**

Brain diffuse gliomas are highly epileptic and infiltrative tumors. Glioma surgery consists of the resection of the tumor core and the maximum of the peritumoral zone, infiltrated by tumor cells, guided by the intraoperative assessment of brain functionality and connectivity. However, its electrophysiological characteristics are poorly characterized.

**Methods:**

We studied the characteristics of electrocorticographic (ECoG) signals, in the context of glioma surgery in awake conditions on 29 patients, using EEG activity sampled on the tumor itself versus on its borders and in healthy areas. We assessed the features of frequency bands and aperiodic components (offset and slope) of ECoG power spectra during awake glioma surgery, according to cortical tumoral versus peritumoral and healthy status.

**Results:**

We found that tumor contacts present a decrease in activity for all the frequency bands except for delta activity, which was increased. Second, the peritumoral cortex was characterized by an increase in relative beta activity and slopes between 20 and 40 Hz. Low cortical tumor cell infiltration was directly correlated with a reduction in the production of physiological brain rhythms. Finally, an automatic classifier based on neural networks allowed the classification of the electrodes based on their power spectrum characteristics.

**Conclusions:**

This intraoperative study shows that ECoG during glioma surgery in awake condition may characterize the peritumoral cortices, key for pathophysiology and therapy, and deepens our knowledge of the effects of tumor cell infiltration on nervous tissue activity. Its assessment during the surgical procedure should better delineation of the cortical areas to be removed.

## Graphical Abstract

**Graphical Abstract:**
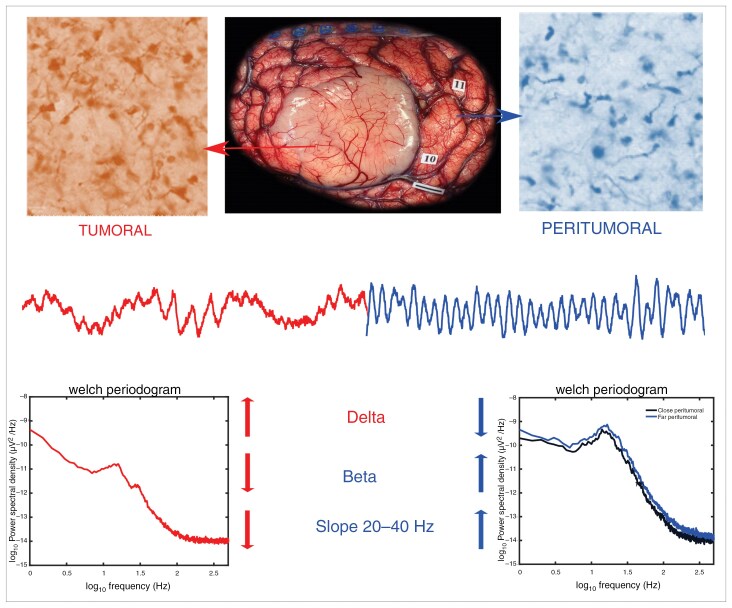

**Key Points**

- Tumoral cortex displays a decrease in activity with an increase in delta frequency.
- Peritumoral contacts present an increase in beta activity and the 20–40 Hz slope.

**Importance of the Study**

Our pilot study on awake electrocorticographic (ECoG) recording of patients undergoing surgery for glioma in awake condition shows that specific features of the power spectrum characterize the tumoral versus peritumoral cortex. Furthermore, peritumoral infiltration by tumor cells is correlated to specific ECoG features. This key but difficult-to-delineate zone surrounding gliomas is mandatory to be removed to improve both epilepsy and oncological outcomes. Our study indicates that ECoG during glioma surgery in awake condition might improve our knowledge of the effects of tumor cell infiltration on nervous tissue behavior and the surgical procedure itself by better delineating the cortical areas to be removed. It paves the way to real-time electrophysiological mapping to better guide the resection, which would impact the patients’ oncological and epileptological outcomes, with larger survivals and improved quality of life.

Gliomas are the most frequent primary brain tumor and are highly epileptogenic. Between 35% and 89% of patients present epileptic seizures at diagnosis.^1,2^ Seizure frequency increases as the tumor progresses, and patients may become resistant to antiseizure medication, which significantly impacts their quality of life.^3^ Multiple mechanisms are implicated in tumor-related epilepsy, including glutamate and GABA signaling impairment,^4,5,6^ which also affect glioma growth processes. Moreover, neuronal activities contribute to glioma growth,^7–11^ and interactions between the tumor and surrounding cortex provide feedback on tumor growth.^12^

Gliomas are diffuse tumors formed by a macroscopic core, identifiable on MRI, surrounded by sparser tumor cell infiltration, extending beyond MRI-defined limits,^13^ known as the peritumoral tissue. The oncological and epileptological processes converge at this interface between healthy and pathological tissues. Tumor cells spread in the peritumoral tissue,^13^ and epilepsy discharges arise at more than 1.5–3 cm from the tumor core.^4,14,15^ The peritumoral cortex also produces high-frequency oscillations,^16,17^ reflecting a hyperexcitability state. Such epileptic activities have been shown to colocalize with peritumoral infiltration in human postoperative tissues.^4^

Peritumoral tissue identification and management are therefore critical during surgery, the first-line glioma treatment. Maximal removal of tumor cells requires intraoperative functional assessment of peritumoral areas, which warrants prolonged survival and safer surgery.^18^ Such supramaximal resection encompassing isolated glioma cells is performed under awake conditions to identify brain connectivity within the peritumoral cortex and white matter bundles and to achieve a function-based resection.^19^ Brain functions are tested intraoperatively using direct electrical stimulations. Electrocorticography (ECoG) can detect spontaneous interictal epileptic activities and epileptic seizures induced by direct electrical stimulations.^20,21^ The value of the integration of the epileptic activity detected by intraoperative ECoG in the resection is still a matter of debate.^22,23^

However, the characterization of tumoral versus peritumoral areas using electrophysiology gathered limited attention, as tumor detection and delineation are performed by imaging. In recent years, the analysis of the frequency bands of the power spectrum, known as “canonical frequency bands” has shown differences between the tumor and the healthy tissue.^24^ Indeed, the aperiodic components of the power spectrum, describing the non-oscillatory activity in the power spectrum, distinct from periodic oscillations and reflecting underlying neural noise or baseline activity, such as the offset and slope, have been studied in several pathologies, such as schizophrenia or Parkinson disease, but less is known about gliomas and their surrounding tissue.^25,26^ In particular, the slope of the power spectrum is thought to be a marker of the excitation-inhibition balance,^26–28^ known to be key in epileptic and glioma growth processes.^5^

This study aims to identify in vivo electrophysiological biomarkers recorded intraoperatively using ECoG to better characterize the peritumoral cortex and to delimitate the extension of the peritumoral area during awake surgery by studying the characteristics of the power spectrum (Figure 1).

**Figure 1.**
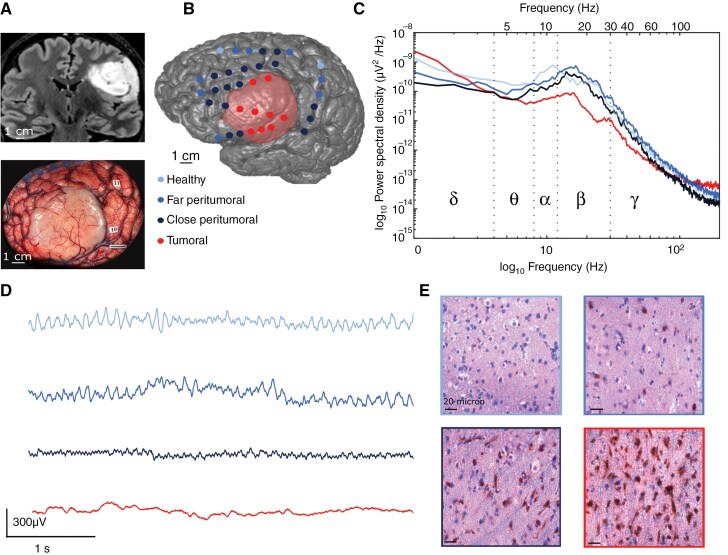
Intraoperative ECoG recording during Glioma surgery. (A) MRI sagittal view of a left tumor and perioperative picture. (B) 3D volume rendering with the position of the contacts superimposed- tumoral (red), close peritumoral (dark blue), far peritumoral (mild blue) and healthy (pale blue). (C) ECoG traces of the 4 compartments. (D) Welch periodogram of 4 contacts, one from every location. (E) Immunohistochemistry (IDH1R132H labeling) and hematoxylin staining showed a healthy biopsy, a low infiltrated (<50 tumoral cells/250 µm^2)^, a highly infiltrated (>50 tumoral cells/250 µm^2^) and a tumoral biopsy (from an abnormal MRI area).

## Materials and Methods

### Patients

We retrospectively analyzed ECoG data from 29 adult glioma patients recorded during awake surgery between November 2020 and March 2023 at Sainte-Anne Hospital and Pitié-Salpêtrière Hospital (Paris, France). The presence of epilepsy, tumor location, WHO tumoral grade, and IDH-mutation status were assessed. This study was approved by the Comité d’Evaluation et d’Ethique de l’INSERM - IRB00003888 (approval n°21-864). Subjects’ consent was obtained according to the Declaration of Helsinki.

### Surgery

As recommended by the EANO and SNO guidelines, the primary goal of diffuse glioma surgery is to achieve maximal safe resection.^29,30^ Patients with diffuse gliomas located within or near functional areas underwent surgery using a functional-based approach, employing the asleep–awake-asleep technique using a previously described methodology.^21,31^ The craniotomy exposed the lesion in addition to 2 to 3 cm of peritumoral cortex around the lesion to ensure a positive functional brain mapping.^32^

### ECoG Recordings

ECoG is routinely used in glioma awake surgery in both hospitals (Sainte-Anne and Pitié-Salpêtrière), for safety reasons to allow the intraoperative identification of spontaneous interictal discharges and after-discharges or epileptic seizures induced by direct electrical stimulations. Once the patients were awake and able to perform the intraoperative functional testing, the neurosurgeon placed the sterile electrodes directly in contact with the cortex (Figure 1). The same position was kept for at least 2 minutes. Then, the electrode was moved to an adjacent area to map the exposed cortex. The recording did not modify the surgical procedure. A 6 × 1 strip and a 4 × 2 grid (DIXI Medical) were used, depending on the morphology and size of the craniotomy. The electrodes’ diameter was 4 mm, and the interelectrode space was 10 mm. A needle reference and ground electrodes were placed in the scalp, next to the craniotomy. Data were acquired using an Atlas System (Neuralynx) or an ISIS Inomed amplifier using a 20 KHz sampling rate. Only the presurgical mapping before electrical stimulation was analyzed.

### Electrodes Classification

The neurosurgeon identified the anatomic location of the tumor by macroscopic visual analysis, MRI images, and echography. The position of the electrodes was noted, and pictures of every position were taken. For subcortical lesions, tumor margins were defined based on FLAIR hyperintensity. 3D-MRI volume rendering was obtained using 3D-Slicer reconstruction on FLAIR MRI sequences (https://www.slicer.org, Brigham and Women’s Hospital). The intraoperative pictures with the electrode position were merged into the 3D-MRI reconstructions, matching the gyral patterns, to measure the distance between the tumor border on the FLAIR sequence and the contacts. Therefore, an electrode was classified as: “tumoral” if it was contacting an MRI-positive area and/or if it had a suspicious appearance macroscopically; “close peritumoral” if placed at <15 mm from the tumor border; “far peritumoral” if placed between 16 and 30 mm from the tumor margin; and “healthy” if set at more than 31 mm. For deep lesions, no tumoral electrodes were designated, as these contacts did not directly overlay the tumor. Such 1.5/3 cm limits of the peritumoral area were defined according to previous studies on peritumoral infiltration.^13^

### Immunostaining

A total of 26 brain biopsies from the peritumoral security margin of 15 patients were obtained during the surgery of IDH-mutated gliomas. They were obtained from cortical areas in direct contact with a recording electrode located in the planned resection area. The average distance from the biopsies to the tumor was 8.1 ± 6.25 mm (range 0–20.28 mm). Slices were cut using a vibratome (Leica VT1200S) in 400 µm. Then, they were fixed in formalin-zinc (Diapath) for 24–72 hours. They were embedded on agar to be resliced in 50 µm sections. After 10 minutes of H_2_O_2_ application to block internal peroxidase, the slices were heated at 100 °C in Tri-Sodium citrate buffer 10 mM for antigen retrieval over 30 minutes. Slices were permeabilized with PBS 0.25% for 1 hour, then they were incubated overnight with a mouse primary antibody against IDHR321H mutation (1:100, Quartett). Immunochemistry was performed with DAB reagent (Abcam) and counterstained with hematoxylin.

### Data Preprocessing

The raw signal from all channels for each recording was visualized using our graphical interface developed in Matlab (The Mathworks). A monopolar setup was used taking as a reference the needle electrode of the acquisition system, and the signals were forward–backward filtered (between 0.1 and 50 Hz) using a Chebyshev type II bandpass IIR filter. Noisy channels were removed and the signal segments with artifacts were excluded. The remaining data segments were subsampled (2000 Hz) and classified according to their position in the brain (tumor, close, far peritumoral, and healthy).

### Power Spectrum Analysis

To assess the power distribution into the different frequency components of ECoG signals, power spectral density (PSD) was computed over the raw data using the Welch method in each signal segment (Hanning window of *n* = 2* Sample Rate with an overlap of 50%; Figure 1). Afterward, the electrical noise peaks (50 Hz and its harmonics) were removed from the PSD and the total power spectrum was computed by summing the remaining points. Absolute and relative power^33^ were calculated in the following frequency bands: delta (0.5–4 Hz), theta (4–8 Hz), alpha (8–12 Hz), beta (1229 Hz), low gamma (30–45 Hz), and high gamma (55–100 Hz). The absolute power values were obtained by summing the PSD into each frequency band, while the relative power by dividing this sum by the total spectrum power. To compare data from different patients, power values were normalized using the maximum power value from each patient.

### Aperiodic Components Frequency Analysis

To study the 1/f-like aperiodic component of the PSD, we estimate the offset and slope. To compute these values, the FOOOF model was used.^34^ It parameterizes the power spectra as a combination of periodic and aperiodic components. Power spectra were parameterized across the frequency ranges 1–200, 20–30, 20–40, 30–45, 40–60, and 60–120 Hz.

### Phase Locking Value

We studied the phase synchrony over contacts to measure connectivity between the different compartments. These phase interactions were accessed using the phase locking value (PLV). First, raw signals were filtered in 6 frequency bands (0.5–4, 4–8, 8–12, 12–30, 30–45, and 55–100 Hz) using a Chebyshev type II bandpass IIR filter. Then, for each one the phase of the signal was calculated, using the Hilbert transform in each frequency band. Afterward, the phase difference and PLV between the contacts recorded simultaneously in the operating room but covering different tissue areas were computed.

### Statistics

Data were expressed as mean and SD for continuous variables and percentages for categorical variables. We used GraphPad software to analyze the results. The spectral features were compared between all 4 different positions using a Kruskall–Wallis test. A post hoc Dunn’s test was applied to correct for multiple comparisons to mitigate the risk of false positives arising from multiple comparisons. We considered significant values of *P* inferior to .05. Astrocytoma and oligodendroglioma patients were compared using a Mann–Whitney test. A Spearman correlation test was used to compare the distance in mm and the tumoral infiltration with all the features.

### Algorithm Classifier

Two different models of machine learning classifying between tumoral, close, far peritumoral, and healthy tissue, were implemented, based on k-nearest neighbors (kNN) and support vector machines (SVM). The classifiers used the features extracted from the power spectrum analysis as inputs. A total of 38 features were used for classification purposes, 12 were periodical features (absolute and relative power in 6 frequency bands) and 7 were aperiodical (offset of the power spectrum and slope in 6 different frequency bands). The selected features were used in a normalized and not normalized version. From the 710 electrodes, 121 were placed over the tumor, 456 were placed in the peritumoral area (243 close and 212 far), and 134 were placed over healthy tissue. The electrodes were randomly split into 2 groups, 70% of the data was used for training and validation (*n* = 496), and the remaining 30% for testing the classification algorithms (*n* = 212). On the training contacts, cross-validation with 5 folds was applied, which avoids overfitting and decreases the dependency of the results on the specific data partition. Different parameters of k-NN and SVM classifiers were optimized during the training/validation phase. Finally, the testing group was used to evaluate the optimal models on new data. Two different classification strategies were performed. The first one uses 4 classes: tumoral, close peritumoral, far peritumoral, and healthy contacts. The second one, and following the literature, uses 2 classes: tumoral and non-tumoral.

## Results

We obtained electrophysiological data from 718 contacts in 29 glioma subjects recorded with ECoG during awake surgery. Most cases were astrocytoma IDH-mutant (19/29; 65.51%), followed by oligodendroglioma IDH-mutant-1p/19q-codeleted (6/29; 20.68%), glioblastoma IDH-WT (2/29; 6.89%), and 2 non-typable gliomas (Supplementary Table 1). WHO grade 3 was the most represented grade (13/29; 44.82%) followed by WHO grade 2 (11/29; 37.93%) and WHO grade 4 (5/29; 17.24%). Most patients (27/29; 93.1%) presented with epileptic seizures at diagnosis, and none of them were drug-resistant before surgery. None of the subjects under study experienced early postoperative seizures, nor did they encounter surgical complications such as bleeding, infection, or the need for an additional surgical procedure. The most frequent tumor location was frontal (19/29, 65.5%), followed by temporal (5/29, 17.2%), and insular (5/29, 17.2%). Unilobar location (21/29, 72.4%) was more frequent than multilobar implication (8/29, 27.6%). Cortical involvement (18/29, 62.1%) was more frequent than pure subcortical location (11/29, 37.9%). From the 718 electrodes, 126 were placed over the tumor, 456 were placed in the peritumoral area (243 close, 212 far), and 136 were placed over healthy tissue. Of all the 29 patients, 7 (24.1%) presented spontaneous interictal discharges (IIDs). IIDs were not detected on electrodes placed over tumor tissue. The mean distance from the tumoral border to the IIDs was 17 mm (range 2.89–32.65 mm).

### Assessment of ECoG Frequency Bands in Glioma Cortical Compartments

**Figure 2.**
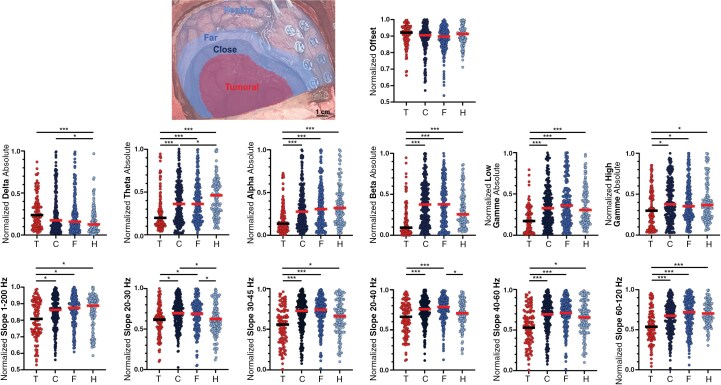
Frequency bands and aperiodic components analysis of tumoral and peritumoral cortices. (A) Intraoperative picture showing the limits of the 4 compartments: tumoral (red), close peritumoral (dark blue), far peritumoral (mild blue), and healthy (pale blue). (B) Plot showing the normalized spectrum features: delta, theta, alpha, beta, low gamma, high gamma, offset, slope 1–200, 20–30, 20–40, 30–45, 40–60, and 60–120 Hz. * *P* < .05, *** *P* < .001.

We first looked for differences in the frequency signature between peritumoral tissue, tumor, and healthy contacts (Table 1). Tumoral contacts were characterized by a reduction in the PSD of all the frequency bands in absolute and relative values, except for the delta band, in which an increased activity was observed (0.28 ± 0.16 in absolute and 0.72 ± 0.14 in relative values), compared to close peritumoral (0.29 ± 0.23 in absolute and 0.65 ± 0.17 in relative, *P* = .43 and .02), far peritumoral (0.27 ± 0.2 in absolute and 0.63 ± 0.16 in relative, *P* = .11 and .01) and healthy contacts (0.2 ± 0.14 in absolute and 0.64 ± 0.16 in relative, *P* < .001 and .01; Figure 2).

**Table 1.** Mean Values of the Power Spectrum Features in the Tumoral, Close Peritumoral, Far Peritumoral, Healthy Compartments, Assessed According to Their Macroscopic/MRI Location From Tumor Limits, and Mean Values in the Biopsies of Highly Infiltrated, Low Infiltrated and Healthy Tissues

Feature	Tumoral	Close	Far	Healthy	*P*	Highly infiltrated	Low infiltrated	Healthy	
δ absolute	0.28 ± 0.16	0.29 ± 0.23	0.27 ± 0.2	0.19 ± 0.14	**.002**	0.22 ± 0.35	0.33 ± 0.32	0.47 ± 0.34	0.18
θ absolute	0.27 ± 0.2	0.38 ± 0.22	0.39 ± 0.2	0.46 ± 0.21	.16	0.13 ± 0.08	0.31 ± 0.26	0.43 ± 0.22	**0.04**
α absolute	0.19 ± 0.13	0.34 ± 0.21	0.37 ± 0.2	0.38 ± 0.21	**<.0001**	0.35 ± 0.19	0.3 ± 0.22	0.55 ± 0.19	0.05
β absolute	0.17 ± 0.15	0.41 ± 0.24	0.42 ± 0.24	0.33 ± 0.19	**<.0001**	0.45 ± 0.24	0.35 ± 0.25	0.48 ± 0.21	**0.003**
Low gamma absolute	0.21 ± 0.13	0.38 ± 0.22	0.42 ± 0.24	0.37 ± 0.21	**<.0001**	0.37 ± 0.33	0.37 ± 0.26	0.49 ± 0.3	0.23
High gamma absolute	0.34 ± 0.21	0.43 ± 0.22	0.42 ± 0.21	0.44 ± 0.21	**.006**	0.3 ± 0.37	0.41 ± 0.3	0.47 ± 0.28	0.27
δ relative	0.73 ± 0.14	0.65 ± 0.17	0.63 ± 0.18	0.64 ± 0.16	**.002**	0.76 ± 0.23	0.68 ± 0.19	0.71 ± 0.2	0.92
θ relative	0.32 ± 0.19	0.38 ± 0.22	0.36 ± 0.22	0.39 ± 0.24	**<.0001**	0.18 ± 0.16	0.42 ± 0.36	0.21 ± 0.13	0.25
α relative	0.24 ± 0.15	0.4 ± 0.24	0.41 ± 0.25	0.36 ± 0.22	**<.0001**	0.5 ± 0.36	0.46 ± 0.31	0.28 ± 0.12	**0.001**
β relative	0.22 ± 0.19	0.45 ± 0.28	0.45 ± 0.26	0.37 ± 0.25	**<.0001**	0.56 ± 0.42	0.44 ± 0.33	0.2 ± 0.13	**0.04**
Low gamma relative	0.2 ± 0.14	0.35 ± 0.23	0.35 ± 0.23	0.33 ± 0.23	**<.0001**	0.39 ± 0.33	0.41 ± 0.3	0.25 ± 0.25	0.32
High gamma relative	0.29 ± 0.19	0.36 ± 0.23	0.35 ± 0.21	0.33 ± 0.23	**<.0001**	0.17 ± 0.2	0.43 ± 0.34	0.15 ± 0.08	**0.04**
Offset	0.89 ± 0.07	0.86 ± 0.11	0.87 ± 0.07	0.91 ± 0.05	**.03**	0.86 ± 0.18	0.95 ± 0.03	0.92 ± 0.05	0.56
Slope 1–200 HZ	0.81 ± 0.09	0.85 ± 0.07	0.85 ± 0.07	0.85 ± 0.08	**.001**	0.81 ± 0.11	0.78 ± 0.12	0.86 ± 0.09	0.32
Slope 20–30 HZ	0.62 ± 0.14	0.69 ± 0.14	0.68 ± 0.15	0.62 ± 0.15	**<.0001**	0.78 ± 0.23	0.81 ± 0.16	0.51 ± 0.24	**0.01**
Slope 20–40 HZ	0.62 ± 0.18	0.74 ± 0.85	0.77 ± 0.11	0.71 ± 0.12	**<.0001**	0.76 ± 0.19	0.79 ± 0.16	0.74 ± 0.08	0.5
Slope 40–60 HZ	0.61 ± 0.17	0.71 ± 0.16	0.73 ± 0.16	0.68 ± 0.17	**<.0001**	0.82 ± 0.27	0.65 ± 0.19	0.81 ± 0.16	0.46
Slope 30–45 HZ	0.53 ± 0.21	0.69 ± 0.17	0.71 ± 0.14	0.65 ± 0.16	**<.0001**	0.8 ± 0.27	0.69 ± 0.18	0.74 ± 0.09	0.35
Slope 60–120 HZ	0.56 ± 0.16	0.65 ± 0.16	0.69 ± 0.17	0.72 ± 0.13	**<.0001**	0.83 ± 0.2	0.6 ± 0.26	0.69 ± 0.25	0.49

Mean values of the power spectrum features in the tumoral, close peritumoral, far peritumoral, and healthy compartments, assessed according to their macroscopic/MRI location from tumor limits, and mean values in the biopsies of highly infiltrated, low infiltrated, and healthy tissues (significant *P* values in bold).

The close peritumoral contacts were characterized by an increase in beta relative values at the close peritumoral level (0.45 ± 0.28) and far peritumoral contacts (0.45 ± 0.26) compared to both tumoral (0.22 ± 0.19, *P* < .001 and <.001) and healthy contacts (0.37 ± 0.25, *P* = .24 and .05). The values were not different between close and far peritumoral contacts (*P* = .62; Figure 2).

### Assessment of ECoG Aperiodic Components in Glioma Cortical Compartments

We next examined how the aperiodic components of the power spectrum changed according to the location. We analyzed the offset and different sets of slopes (from 1 to 200, 20 to 40, 30 to 40, 30 to 45, 40 to 60, and 60 to 120Hz) to determine which bandwidths were more useful to differentiate tumoral, peritumoral, and healthy compartments (Table 1). Tumoral contacts presented a decrease of the 1–200 Hz slope (0.81 ± 0.09) compared to close peritumoral (0.85 ± 0.07, *P* = .02), far peritumoral (0.86 ± 0.07, *P* = .01) and healthy contacts (0.85 ± 0.08, *P* = .01; Figure 2). The 20–30 Hz slope showed specific patterns at peritumoral locations. For close peritumoral positions, we recorded an increase (0.77 ± 0.14) compared to tumoral (0.62 ± 0.14, *P* = .01) and healthy contacts (0.62 ± 0.15, *P* = .02). Far peritumoral contacts also presented a slope increase (0.68 ± 0.15) compared to tumoral (*P* = .01) and healthy contacts (*P* = .01). No differences were observed between close and far peritumoral contacts (*P* = .72). At the far peritumoral level, the 20–40 Hz slope (0.77 ± 0.11) differed from healthy ones (0.708 ± 0.12; *P* < .001; Figure 2).

### Malignancy Grade and IDH-mutated Status Influence the Power Spectrum

We then examined whether the malignancy grade or IDH-mutation status influenced our results. WHO grade 2 tumors exhibited an increase in beta relative values at close and far peritumoral levels (0.41 ± 0.34 and 0.38 ± 0.3) compared to tumoral (0.1 ± 0.15, *P* < .001 for both) and healthy contacts (0.3 ± 0.24, *P* = .02). WHO grade 3 tumors were also characterized by higher beta relative values in close (0.39 ± 0.32) and far peritumoral regions (0.38 ± 0.26) compared to tumoral (0.21 ± 0.26, *P* < .001 for both) and healthy contacts (0.21 ± 0.26, *P* = .01 for both). In WHO grade 4 gliomas, higher beta relative values were observed in close peritumoral (0.48 ± 0.29), far peritumoral (0.59 ± 0.28), and healthy regions (0.6 ± 0.25) compared to the tumor (0.32 ± 0.22, *P* = .02, .03, and .02). However, there were no significant differences between the peritumoral areas and the healthy regions (*P* = .83 and .99; Supplementary Table 2).

Regarding the aperiodic components, WHO grade 2 gliomas demonstrated an increase in the 40–60 Hz slope at the peritumoral (0.7 ± 0.18 for close and 0.74 ± 0.17 for far) and healthy regions (0.7 ± 0.19) compared to the tumor (0.65 ± 0.19; *P* = .01, <.001, and .01). WHO grade 3 gliomas displayed higher 20–30 Hz slope values in the close (0.71 ± 0.16) and far peritumoral regions (0.71 ± 0.2) compared to the tumor (0.57 ± 0.17; *P* = .01 for both) and healthy regions (0.54 ± 0.23; *P* < .001 for both). Lastly, WHO grade 4 gliomas showed an increase in the 20–40 Hz slope in the peritumoral (0.51 ± 0.21 for close and 0.67 ± 0.19 for far) and healthy regions (0.75 ± 0.14) compared to the tumor (0.51 ± 0.21; *P* = .02, <.001, and .01; Supplementary Table 2).

After excluding the 2 glioblastoma-IDH-WT patients, the analysis of the remaining 27 IDH-mutant patients yielded results similar to the overall patient analysis. These patients also showed increased beta values in the close (0.41 ± 0.3) and far peritumoral cortex (0.36 ± 0.27) compared to the tumor (0.12 ± 0.13; *P* < .001 for both) and the healthy areas (0.29 ± 0.23; *P* = .02 and .8). The 20–40 Hz slope followed a similar pattern, with higher values in the peritumoral regions (0.69 ± 0.17 close and 0.77 ± 0.16 far peritumoral) compared to the tumor (0.69 ± 0.17; *P* = .03 and .01) and the healthy cortex (0.29 ± 0.23; *P* = .01 and <.001; Supplementary Table 3).

### Intra- and Inter-compartment Functional Connectivity Features

**Figure 3.**
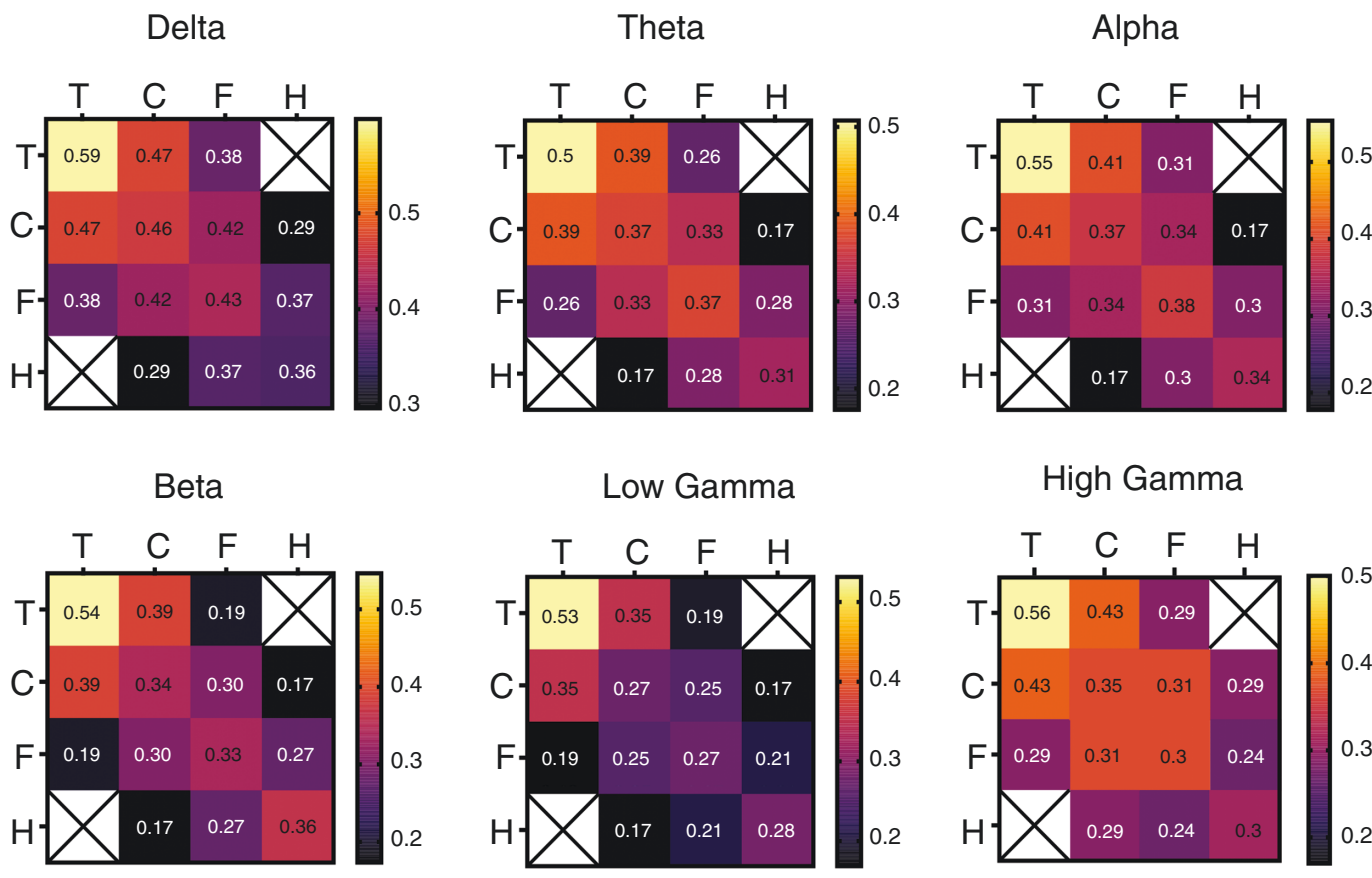
Phase Locking Value (PLV) of tumoral, close peritumoral, far peritumoral, and healthy compartments for each frequency band. PLV matrix represented in a heat map, with the mean values inside, for the different frequency ranges (from delta to high gamma).

We then assessed the intra- and inter-compartment connectivity using the PLV. PLV was higher between tumoral contacts (0.59 ± 0.27), followed by close peritumoral (0.46 ± 0.23), far peritumoral (0.43 ± 0.22), and healthy ones (0.36 ± 0.17) in the delta range, but also theta (0.5 ± 0.25 for tumoral, 0.3 ± 0.18 close peritumoral, 0.37 ± 0.19 far peritumoral, and 0.32 ± 0.17 inside the healthy compartment), alpha (0.55 ± 0.25 intratumoral, 0.37 ± 0.19 for close peritumoral, 0.38 ± 0.2 for far peritumoral, and 0.34 ± 0.2 for healthy), beta (0.54 ± 0.25 intratumoral, 0.34 ± 0.2 for close peritumoral, 0.34 ± 0.19 for far peritumoral, and 0.36 ± 0.18 in healthy), low gamma (0.53 ± 0.23 intratumoral, 0.27 ± 0.18 for close peritumoral, 0.27 ± 0.19 for far peritumoral, and 0.28 ± 0.17 for healthy), and high gamma ranges (0.56 ± 0.2 intratumoral, 0.35 ± 0.18 for close peritumoral, 0.3 ± 0.18 for far peritumoral, and 0.3 ± 0.19 for healthy; Figure 3).

### Distance From the Tumor Border Influences the Power Spectrum

We then studied if there was a correlation between the distance of the electrode from the tumor border and the changes in the power spectrum features. A 2-tailed Spearman correlation test did not show a strong correlation between the distance and the different features. The strongest association was found with offset (R = 0.28, IC 95% 0.19–0.36, *P* < .001). Those results suggest that the peritumoral area does not follow a radial distribution but rather an eccentric one, possibly sculpted by glioma cell infiltration. We therefore performed a correlation analysis between the ECoG signal and quantified tumor cell infiltration in the tissue directly sampled during surgery.

### Peritumoral Quantified Infiltration by Tumor Cells Impacts ECoG Frequency Patterns

To go further in our analysis, we correlated the intensity of peritumoral infiltration with the power spectrum. To do so, we measured the density of tumoral cells in 26 fragments from 15 patients with IDH-mutated gliomas, obtained from the security margin during the surgery, for which their exact placement was noted in contact with a recorded channel. We performed immunohistochemistry using R132H-IDH labeling to quantify the number of infiltrative tumor cells (tumor cells/250 µm^2^). The number of tumor cells per area was correlated with the different features of the spectrum previously calculated (Table 1).

**Figure 4.**
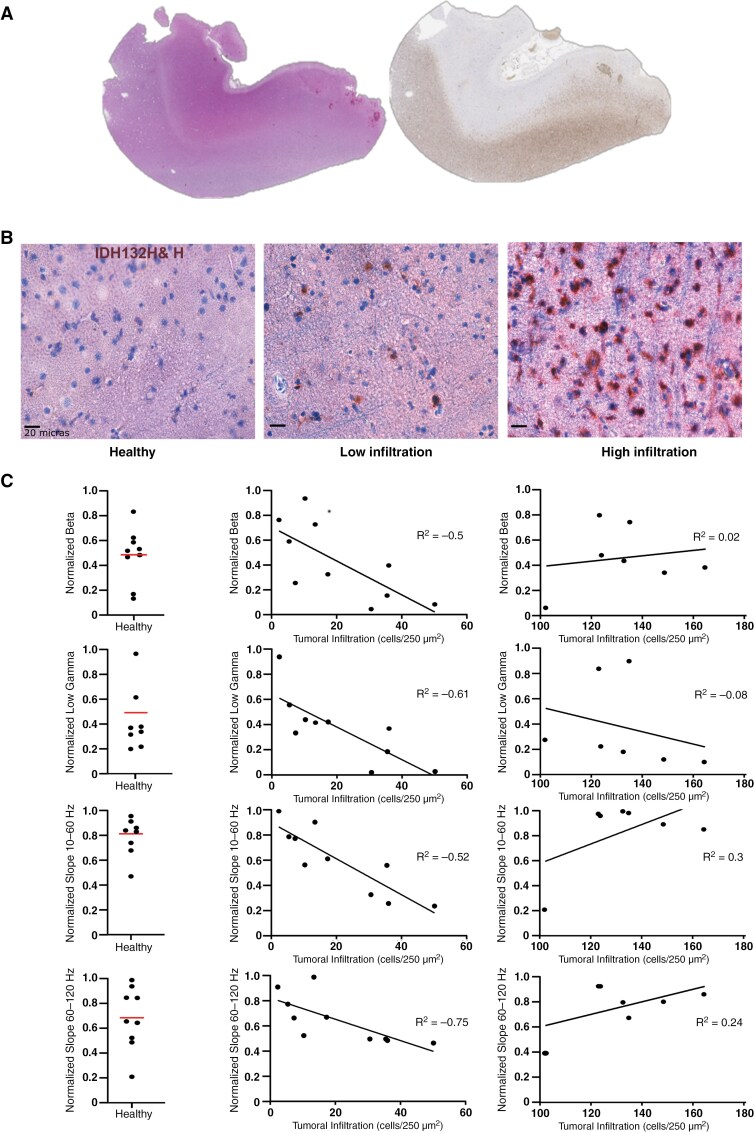
Correlation between glioma cells infiltration and ECoG frequency features. (A) Mirror slices immunohistochemistry using IDH1R132H labeling and hematoxylin and eosin. (B) Immunohistochemistry (IDH1R132H labeling and hematoxylin) shows tumor cells (dark brown) presence: healthy without tumoral cells, low infiltrated (<50 tumoral cells/250 µm^2^), and highly infiltrated (>50 tumoral cells/250 µm^2^). (C) Correlation between beta frequencies, low gamma, slope 40–60 and 60–120 Hz in healthy, low infiltrated, and highly infiltrated biopsies.

For the low infiltrated biopsies (<50 tumor cells/250 µm^2^), we found that the higher the peritumoral infiltration was, the lower the beta absolute values were (R = −0.5, *P* = .04; Figure 4). We also found a similar tendency towards lower values on low gamma activity (R = −0.61, *P* = .02). and in the slope from 40 to 60 Hz (R = −0.52, *P* = .03) and from 60 to 120 Hz (R = −0.75, *P* = .01). The other features did not show any correlation (Table 1). Healthy and highly infiltrated (>50 tumor cells/250 µm^2^) biopsies did not show the same behavior (Figure 4).

### ECoG Pattern Differences Between Astrocytomas-IDH-mutant and Oligodendrogliomas-IDH-mutant-1p19qcodeleted

**Figure 5.**
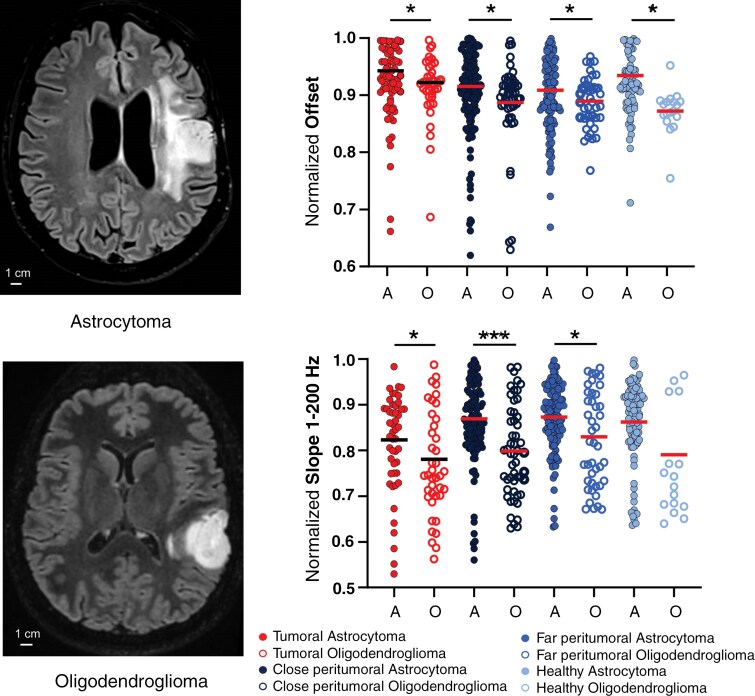
ECoG differential frequency features between oligodendrogliomas IDH-mutant-1p19qcodeleted and astrocytomas IDH-mutant. MRI axial view of an astrocytoma and an oligodendroglioma. Plot comparing the offset and 1–200 Hz slope between astrocytomas (filled dot) and oligodendrogliomas (empty dot). * *P* < .05, *** *P* < .001.

We analyzed whether astrocytomas IDH-mutant and oligodendrogliomas IDH-mutant-1p19qcodeleted presented differential frequency bands and aperiodic component characteristics. Only differences in the offset and 1–200 Hz slopes were found (Figure 5; Supplementary Table 4).

### Algorithm Classifier of Peritumoral Versus Tumoral and Healthy Cortical Recordings

We analyzed ECoG recordings characteristics from 708 contacts sampling areas classified as tumoral, peritumoral (close and far), and healthy using 38 periodical and aperiodical features. A partition of 70% of data was used for training and validation purposes. The remaining 30% was used for testing the algorithm. Two different machine learning models were implemented, k-nearest neighbors (kNN) and SVM. After the cross-validation (k = 5), we obtained the best performance for an ensemble of 30 kNN learners with a 19-neighbor subspace, and for the cubic kernel function in the SVM classifier. The confusion matrices for both classifiers are shown in Supplementary Data, during validation and testing.

From the confusion matrices, the performance was measured in terms of precision, recall, and F1 score (Supplementary Figure 1). The results of the testing phase for the classification of 4 classes depended on the classifier. The model kNN presented a precision of 51.9%, a recall of 51.8%, and an F1-score of 51.8%. On the other hand, SVM presented a precision of 50.2%, a recall of 48.1%, and an F1-score of 49.1%. The chance level for 4 classes is 25%, thus the proposed algorithms were well suited for classifying the 4 classes.

Furthermore, we observed in the confusion matrices that the adjacent regions presented a higher number of false predictions. For this reason, we performed a classification of only 2 classes: tumoral and healthy tissue. The performance metrics obtained during testing for the ensemble kNN model were precision of 69.1%, recall of 71.9%, and F1-score of 70.5%. On the other hand, the SVM classifier presented a precision of 68%, recall of 64.6%, and F1-score of 66.3%.

## Discussion

In this study, we characterize the electrophysiological features of peritumoral cortices by comparing, using intraoperative ECoG in awake patients, the frequency and aperiodic component of the power spectrum between the glioma, peritumoral, and healthy cortex. Our study highlights 2 main findings: the peritumoral and tumoral cortex present different power spectrum features, and peritumoral infiltration alters the buildup of physiological brain rhythms.

Glioma peritumoral tissue scarcely displays IIDs in patients with well-controlled tumor-related epilepsy (7/29). In pharmacoresistant patients, the incidence of IIDs has been reported to be higher, reaching 80% of patients.^15^ Epileptic activities may require more time to emerge after anesthesia. Additionally, the 4 different tissue types may experience varying rates of anesthetic dissipation, which could also influence the timing and presence of such activity. When present, the IIDs recorded in our study did not originate from the tumor itself but from the peritumoral cortex at an average distance of 17 mm from the tumor’s border. This finding corroborates previous studies suggesting that epileptic activities arise from peritumoral tissue.^4,14^ Furthermore, a previous study from our group had colocalized the presence of IIDs ex vivo with regions of pronounced peritumoral infiltration. This investigation also demonstrated that IIDs were never recorded from macroscopic tumor slices.^4^

Our ECoG recordings reveal an augmented beta activity within the peritumoral tissue and an increase in the 20–40 Hz slope, with no significant differences between close and far peritumoral contacts. This increase was not dependent on the malignancy grade nor on the presence of IDH mutation. This increase in beta activity at the peritumoral level is a novel observation. As proposed by Gaetz^35^ and colleagues, beta power may correlate with levels of GABA inhibition. We hypothesize that the enhanced beta power at the peritumoral level may reflect a dysregulation of neuronal activity, likely linked to GABA^4^ and glutamate signaling impairment,^36^ culminating in hyperexcitability within that region. Synaptic modifications and rewiring that occur in the peritumoral regions as functions are progressively displaced by tumor infiltration may also account for such increased beta activity. During functional mapping, it is common to observe that functions are located very close to the tumor border.

On the other hand, the tumoral compartment is characterized by an increase in delta activity accompanied by reduced activity across the other frequency bands. This suppression of brain rhythms, except for delta activity, has also been reported at the tumor level in a study of 16 glioma ECoGs.^24^ Previous EEG studies on tumor patients have consistently observed delta waves at the tumor site.^37–39^ While initially attributed to increased intracranial pressure, subsequent research suggested that delta activity might arise due to peritumoral edema^40^ or structural remodeling in peritumoral areas.^41^ Increased PLV in the tumor further identified in our study stress such rearrangements. The increase in delta activity is thought to indicate reduced cortical inhibition stemming from GABA dysfunction.^42^ Regarding the suppression of brain rhythms at the tumor level, assuming gamma activities are a proxy for neuronal spiking,^43,44^ the decline in gamma activity at the tumor level is in line with the drastic reduction in neurons within this compartment.

Regarding the aperiodic components of the power spectrum, we observed steeper slopes between 20 and 40 Hz at the peritumoral level. Deciphering the underlying mechanism for this increase is beyond the scope of this work. Yet some authors propose that steeper slopes signify an imbalance in the excitation-inhibition equilibrium.^27^ Given the heightened excitability in the peritumoral tissue, it is plausible that this excitation-inhibition balance is disrupted. A similar elevation in peritumoral excitability was observed in vivo using ECoG in tumor-related epilepsy glioma patients, with increased fast activities up to 100 Hz^16^ and HFO rates.^17^ Another ECoG study on glioma patients also reported an increased slope at the peritumoral level compared to healthy tissue.^45^ Additionally, a study involving epileptic patients implanted intracranially by StereoEEG showed that the seizure onset zone displayed higher slope values.^28^

Our analysis of the connectivity using the PLV revealed that the tumor compartment presented the highest levels of connectivity. This trend was consistent with a previous study.^24^ Elevated intratumoral connectivity may be attributed to the disruption of standard cortical architecture induced by the tumor mass, which may promote long-range conduction of electrical signals, but also to altered inhibitory processes. It is interesting to note that higher PLVs values may indicate increased neuronal synchronous activity, which may be related to interactions with the observed in the glioblastoma pacemaker-cells phenomenon occurring in the glioma cells network.^46^

Furthermore, astrocytomas and oligodendrogliomas present similar behaviors, only presenting higher values of offset and slope from 1 to 200 Hz in astrocytomas. Since the rest of the features did not reach any statistical signification these differences could be merely statistical outliers.

Given that we employed an arbitrary distance criterion for compartment classification, based on previous studies^13^ we then examined electrode behavior based on the distance to the tumor without applying any threshold. The distance between the macroscopic core and the contact did not correlate with the frequency bands or the aperiodic features. In tumor-related epilepsy, IIDs and the seizure onset zone are typically eccentric to specific areas of the peritumoral tissue, and no radial distribution has ever been noted.^14^ Our data supports that peritumoral tissues follow an eccentric rather than a radial distribution, influenced by glioma cell infiltration along axons and blood vessels. However, the degree of peritumoral infiltration seems to alter brain rhythms, especially beta and low gamma activities, and slopes from 40 to 60 and 60 to 120 Hz.

Following the identification of specific differences in the periodic and aperiodic components based on compartments, we developed a method for automated contact classification based on power spectrum features using machine learning. This approach outperformed random classification, with an F1 score reaching 70.5%, suggesting that electrophysiological markers may hold promise for classifying tumoral, peritumoral, and healthy cortices.

Our preliminary study enrolled a limited number of patients. Analysis was restricted to recordings without major artifacts, which is still an issue in the surgery theater. Most patients had astrocytomas, and due to the reduced practice of awake surgery and ECoG recording in glioblastomas, histological categories were disequilibrated. Our main limitation is the lack of information about the density of peritumoral infiltration for every ECoG channel. We only obtained biopsy samples from 26 cases immediately located beneath a recording contact. Infiltration appears to be rather organized in patches than in radius, according to the comparison of the effects of the distance versus the effective infiltration on electrophysiological patterns. Our study is indeed the first one that performed direct correlations between ECoG features and quantified infiltration. It showed that for low infiltrated tissues, the higher the infiltration was, the lower physiological rhythms were recorded, which, although intuitive, remained to be demonstrated. Concerning the classifier, a limitation in the classification capacity could be data imbalance. As peritumoral contacts were more frequent (241 close and 212 far peritumoral compared to tumoral [121)]or healthy [134]) this can create a bias during the training and lead to a decrease in performance in the test. While our study incorporates an independent test set, external validation using data from other patient cohorts is necessary to confirm the generalizability of these findings and the robustness of the classifier. Moreover, even though the electrodes used present one of the smallest dimensions used in the standard of care, their 4 mm diameter electrodes together with the interelectrode space of 10 mm can limit the spatial resolution. Using smaller electrodes, such as new microECoGs^47^ will help us to overcome that limitation and be more precise.

Our pilot study on awake ECoG recording of patients undergoing surgery for glioma in awake condition shows that tumoral and peritumoral are characterized by specific periodic and aperiodic components of the power spectrum, suggesting that they may be used as potential biomarkers of the peritumoral areas. Furthermore, peritumoral infiltration by tumor cells was correlated to specific ECoG features. This key, but challenging to delineate zone, surrounding gliomas is mandatory to be removed to improve both epilepsy and oncological outcomes and is still not characterized yet.

Our study paves the way to real-time electrophysiological mapping to better guide the resection, which would impact the patients’ oncological and epileptological outcomes. The next steps will be to analyze larger sets of patients with a larger number of biopsies. Then, the use of smaller electrodes using conductive polymers such as PEDOT:PSS^47^ should improve not only the spatial resolution but also allow recording precisely and with accuracy of other kinds of electrophysiological activities, such as multiunit and high-frequency oscillations.^4^ Following the validation of these electrophysiological biomarkers, the electrode classifier algorithm could be implemented in real-time during surgery, facilitating comparisons between ECoG-guided resection and the standard of care. The design of such a clinical trial would not be feasible without first comprehending the underlying electrophysiological phenomena and identifying the most appropriate biomarkers to distinguish between different brain regions.

## Supplementary material

Supplementary material is available online at *Neuro-Oncology* (https://academic.oup.com/neuro-oncology).

noaf082_suppl_Supplementary_Figure_S1

noaf082_suppl_Supplementary_Figure_S2

noaf082_suppl_Supplementary_Materials

## Data Availability

Data are available after a reasonable request to the corresponding author.
