# Supplementary figures and images for: Differentiation of tumor versus peritumoral cortex in gliomas by intraoperative electrocorticography
Source: Neuro Oncol. 2025 Apr 24;27(7):1758–71. doi: 10.1093/neuonc/noaf082 (PMC12417838; doi:10.1093/neuonc/noaf082)

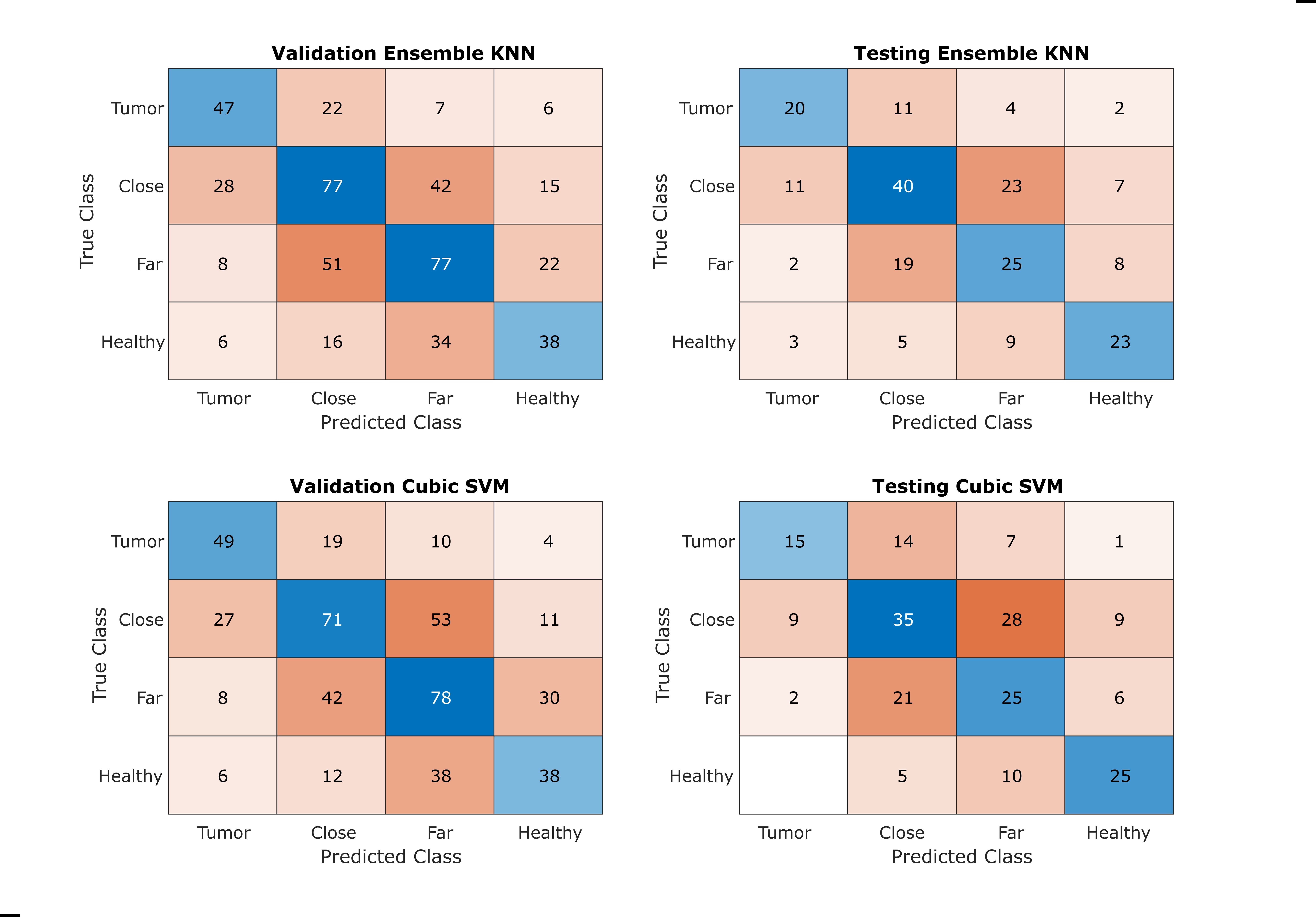

Supplement: noaf082_suppl_Supplementary_Figure_S1 [file noaf082_suppl_supplementary_figure_s1.jpeg]

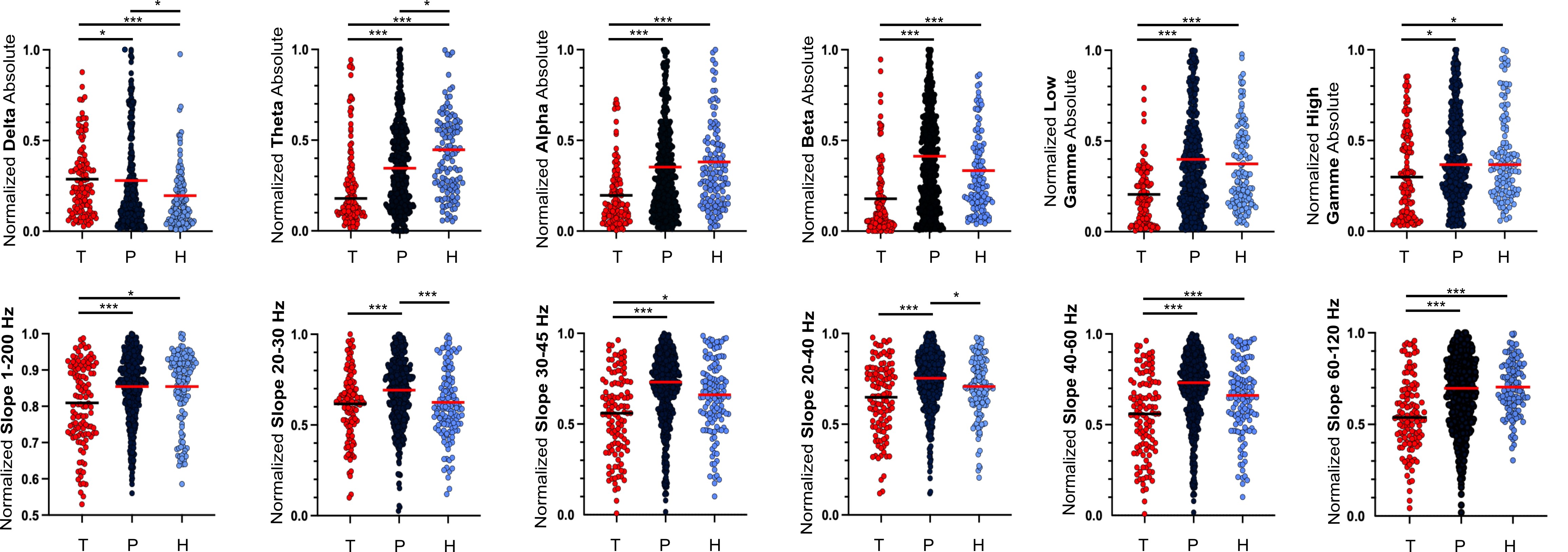

Supplement: noaf082_suppl_Supplementary_Figure_S2 [file noaf082_suppl_supplementary_figure_s2.jpeg]
