# Supplementary material for: Differentiation of tumor versus peritumoral cortex in gliomas by intraoperative electrocorticography
Source: Neuro Oncol. 2025 Apr 24;27(7):1758–71. doi: 10.1093/neuonc/noaf082 (PMC12417838; doi:10.1093/neuonc/noaf082)
Supplement: noaf082_suppl_Supplementary_Materials [file noaf082_suppl_supplementary_materials.docx]

| **Patient** | **Lobe** | **Side** | **Location** | **Pathology** | **IDH** | **IIDs** | **Grade** | | **Epilepsy** | |  |
| --- | --- | --- | --- | --- | --- | --- | --- | --- | --- | --- | --- |
| 1 | Frontal | Right | Cortical | Astrocytoma | R132H | No | | 3 | | Yes | |
| 2  **3** | Frontal  Frontal | Right  Right | Cortical  Cortical | Oligodendroglioma  No typable | R132H  R132H | No  No | | 2  3 | | No  Yes | |
| 4  5  6  7  8  9  10  11  12  13  14  15  16  17  18  19  20  21  22  23  24  25  26  27  28  29 | Frontal  Fronto-parietal  Frontal  Parieto-occipital  Temporo-parietal  Occipital  Frontal  Frontal  Insula  Frontal  Frontal  Insula  Temporo-parietal  Frontal  Frontal  Fronto-temporal  Frontal  Fronto-temporal  Fronto-parietal  Insula  Insula  Frontal  Insula  Fronto-parietal  Temporal  Frontal | Left  Left  Left  Left  Right  Left  Right  Left  Left  Left  Left  Right  Left  Left  Right  Left  Left  Right  Left  Right  Left  Right  Left  Left  Right  Left | Cortical  Cortical  Cortical  Cortical  Subcortical  Cortico-sub  Cortical  Cortical  Subcortical  Subcortical  Subcortical  Subcortical  Cortical  Cortical  Cortical  Subcortical  Cortical  Subcortical  Cortical  Subcortical  Subcortical  Cortical  Subcortical  Cortical  Cortical  Cortical | Oligodendroglioma  Astrocytoma  Astrocytoma  Glioblastoma  Glioblastoma  Astrocytoma  Astrocytoma  Oligodendroglioma  Astrocytoma  Astrocytoma  Astrocytoma  Astrocytoma  Astrocytoma  Astrocytoma  No typable  Astrocytoma  Oligodendroglioma  Astrocytoma  Oligodendroglioma  Astrocytoma  Oligodendroglioma  Astrocytoma  Astrocytoma  Astrocytoma  Oligodendroglioma  Astrocytoma | R132H  R132H  R132H  WT  WT  R132H  R132H  R132H  R132H  R132H  R132H  R132H  R132H  R132H  R132H  R132H  R132H  R132H  R132H  R132H  R132H  R132H  R132H  R132H  R132H  R132H | No  No  Yes  No  No  No  No  No  Yes  No  No  No  No  No  No  No  No  No  Yes  No  Yes  Yes  No  Yes  No  Yes | | 2  3  4  4  4  2  4  3  2  4  2  2  2  2  3  3  2  3  3  3  2  3  2  2  2  3 | | Yes  Yes  Yes  Yes  Yes  Yes  Yes  Yes  Yes  Yes  Yes  Yes  Yes  Yes  Yes  Yes  Yes  Yes  Yes  Yes  No  Yes  Yes  Yes  Yes  Yes | |
|  |  |  |  |  |  |  | |  | |  | |

**Table Supplementary 1 Clinical characteristics of the studied population.**

**Table Supplementary 2 Mean values of the power spectrum features in the tumoral, close peritumoral, far peritumoral and healthy compartments in grade 2, grade 3 and grade 4 patients.**

|  |  | G2 |  |  |  |  | G3 |  |  |  |  | G4 |  |  |  |
| --- | --- | --- | --- | --- | --- | --- | --- | --- | --- | --- | --- | --- | --- | --- | --- |
|  | Tumoral | Close | Far | Healthy | *p* | Tumoral | Close | Far | Healthy | *p* | Tumoral | Close | Far | Healthy | *p* |
| δ Absolute | 0.36 | 0.25 | 0.2 | 0.14 | **<0.001** | 0.23 | 0.32 | 0.32 | 0.22 | 0.95 | 0.19 | 0.3 | 0.34 | 0.23 | **0.02** |
| θ Absolute | 0.25 | 0.4 | 0.36 | 0.48 | **<0.001** | 0.28 | 0.37 | 0.43 | 0.4 | **0.01** | 0.24 | 0.32 | 0.34 | 0.35 | **0.02** |
| α Absolute | 0.23 | 0.4 | 0.34 | 0.4 | **0.01** | 0.17 | 0.35 | 0.36 | 0.34 | **<0.001** | 0.18 | 0.26 | 0.35 | 0.31 | **0.01** |
| β Absolute | 0.14 | 0.42 | 0.38 | 0.24 | **<0.001** | 0.1 | 0.41 | 0.32 | 0.33 | **<0.001** | 0.18 | 0.32 | 0.52 | 0.41 | **<0.001** |
| Low γ Absolute | 0.15 | 0.4 | 0.4 | 0.32 | **<0.001** | 0.19 | 0.37 | 0.4 | 0.42 | **<0.001** | 0.21 | 0.32 | 0.41 | 0.33 | **0.01** |
| High γ Absolute | 0.24 | 0.4 | 0.4 | 0.37 | **0.01** | 0.39 | 0.44 | 0.39 | 0.5 | 0.22 | 0.33 | 0.38 | 0.43 | 0.45 | 0.18 |
| δ Relative | 0.76 | 0.64 | 0.73 | 0.7 | **0.01** | 0.73 | 0.68 | 0.68 | 0.73 | 0.57 | 0.65 | 0.6 | 0.48 | 0.4 | **<0.001** |
| θ Relative | 0.22 | 0.35 | 0.3 | 0.4 | **0.01** | 0.3 | 0.38 | 0.42 | 0.25 | **0.01** | 0.4 | 0.42 | 0.36 | 0.45 | 0.51 |
| α Relative | 0.18 | 0.38 | 0.36 | 0.37 | **0.01** | 0.19 | 0.42 | 0.43 | 0.23 | **<0.001** | 0.37 | O.42 | 0.44 | 0.49 | 0.78 |
| β Relative | 0.1 | 0.42 | 0.38 | 0.26 | **<0.001** | 0.23 | 0.39 | 0.38 | 0.21 | **<0.001** | 0.32 | 0.48 | 0.59 | 0.6 | **<0.001** |
| Low γ Relative | 0.14 | 0.33 | 0.34 | 0.32 | **<0.001** | 0.15 | 0.3 | 0.31 | 0.26 | **0.01** | 0.25 | 0.34 | 0.35 | 0.37 | 0.59 |
| High γ Relative | 0.18 | 0.3 | 0.34 | 0.36 | **<0.001** | 0.34 | 0.35 | 0.3 | 0.33 | 0.46 | 0.32 | 0.36 | 0.36 | 0.51 | 0.21 |
| Slope 1-200 Hz | 0.82 | 0.85 | 0.84 | 0.86 | 0.09 | 0.79 | 0.84 | 0.86 | 0.8 | **0.01** | 0.77 | 0.83 | O.84 | 0.85 | **0.02** |
| Offset | 0.92 | 0.85 | 0.84 | 0.86 | 0.1 | 0.89 | 0.78 | 0.83 | 0.8 | **<0.001** | 0.86 | 0.85 | 0.85 | 0.88 | 0.97 |
| Slope 20-30 Hz | 0.67 | 0.72 | 0.61 | 0.62 | **0.01** | 0.57 | 0.71 | 0.71 | 0.54 | **<0.001** | 0.59 | 0.61 | 0.67 | 0.69 | 0.17 |
| Slope 20-40 Hz | 0.71 | 0.75 | 0.76 | 0.72 | 0.16 | 0.66 | 0.77 | 0.79 | 0.63 | **<0.001** | 0.51 | 0.67 | 0.77 | 0.75 | **<0.001** |
| Slope 40-60 Hz | 0.52 | 0.7 | 0.75 | 0.67 | **<0.001** | 0.62 | 0.74 | 0.75 | 0.57 | **<0.001** | 0.39 | 0.6 | 0.61 | 0.57 | 0.45 |
| Slope 30-45 Hz | 0.65 | 0.7 | 0.74 | 0.7 | 0.1 | 0.57 | 0.73 | 0.8 | 0.7 | **<0.001** | 0.65 | 0.65 | 0.63 | 0.56 | 0.1 |
| Slope 60-120 Hz | 0.65 | 0.68 | 0.7 | 0.75 | 0.43 | 0.53 | 0.66 | 0.72 | 0.64 | **<0.001** | 0.54 | 0.61 | 0.63 | 0.72 | **0.04** |

**Table Supplementary 3 Mean values of the power spectrum features in the tumoral, close peritumoral, far peritumoral and healthy compartments in IDH mutant patients.**

|  | Tumoral | Close Peritumoral | Far Peritumoral | Healthy | *p* |
| --- | --- | --- | --- | --- | --- |
| δ Absolute | 0.29 | 0.28 | 0.26 | 0.17 | **<0.001** |
| θ Absolute | 0.25 | 0.39 | 0.39 | 0.45 | **<0.001** |
| α Absolute | 0.2 | 0.37 | 0.35 | 0.38 | **<0.001** |
| β Absolute | 0.12 | 0.41 | 0.36 | 0.29 | **<0.001** |
| Low γ Absolute | 0.17 | 0.39 | 0.4 | 0.35 | **<0.001** |
| High γ Absolute | 0.32 | 0.43 | 0.4 | 0.42 | **0.01** |
| δ Relative | 0.74 | 0.66 | 0.71 | 0.71 | **0.01** |
| θ Relative | 0.26 | 0.37 | 0.36 | 0.4 | 0.1 |
| α Relative | 0.18 | 0.4 | 0.39 | 0.32 | **<0.001** |
| β Relative | 0.12 | 0.4 | 0.38 | 0.27 | **<0.001** |
| Low γ Relative | 0.15 | 0.32 | 0.33 | 0.3 | **<0.001** |
| High γ Relative | 0.26 | 0.33 | 0.32 | 0.35 | 0.07 |
| Slope 1-200 Hz | 0.8 | 0.86 | 0.85 | 0.84 | **<0.001** |
| Offset | 0.9 | 0.85 | 0.88 | 0.92 | **0.01** |
| Slope 20-30 Hz | 0.67 | 0.72 | 0.7 | 0.59 | **<0.001** |
| Slope 20-40 Hz | 0.69 | 0.76 | 0.77 | 0.68 | **<0.001** |
| Slope 40-60 Hz | 0.57 | 0.72 | 0.75 | 0.65 | **<0.001** |
| Slope 30-45 Hz | 0.61 | 0.72 | 0.77 | 0.7 | **<0.001** |
| Slope 60-120 Hz | 0.59 | 0.67 | 0.71 | 0.7 | **<0.001** |

**Table Supplementary 4 Mean values of the power spectrum features in the tumoral, close peritumoral, far peritumoral and healthy compartments in astrocytoma and oligodendroglioma patients.**

|  |  | Astrocytoma | |  |  |  |  | Oligodendroglioma | | |  | |
| --- | --- | --- | --- | --- | --- | --- | --- | --- | --- | --- | --- | --- |
|  | Tumoral | Close Peritumoral | Far Peritumoral | | Healthy | Tumoral | Close Peritumoral | | Far Peritumoral | Healthy | |  |
| δ Absolute | 0.28 | 0.28 | 0.26 | | 0.18 | 0.31 | 0.22 | | 0.16 | 0.24 | |  |
| θ Absolute | 0.26 | 0.37 | 0.39 | | 0.47 | 0.29 | 0.39 | | 0.38 | 0.34 | |  |
| α Absolute | 0.19 | 0.35 | 0.36 | | 0.35 | 0.24 | 0.35 | | 0.34 | 0.53 | |  |
| β Absolute | 0.13 | 0.39 | 0.39 | | 0.34 | 0.16 | 0.47 | | 0.38 | 0.23 | |  |
| Low γ Absolute | 0.18 | 0.38 | 0.42 | | 0.37 | 0.23 | 0.44 | | 0.37 | 0.29 | |  |
| High γ Absolute | 0.34 | 0.42 | 0.43 | | 0.45 | 0.397 | 0.52 | | 0.36 | 0.34 | |  |
| δ Relative | 0.77 | 0.66 | 0.66 | | 0.68 | 0.72 | 0.68 | | 0.72 | 0.72 | |  |
| θ Relative | 0.36 | 0.35 | 0.35 | | 0.37 | 0.25 | 0.39 | | 0.42 | 0.35 | |  |
| α Relative | 0.27 | 0.41 | 0.39 | | 0.35 | 0.18 | 0.35 | | 0.44 | 0.34 | |  |
| β Relative | 0.20 | 0.45 | 0.42 | | 0.33 | 0.08 | 0.36 | | 0.39 | 0.21 | |  |
| Low γ Relative | 0.23 | 0.34 | 0.35 | | 0.29 | 0.13 | 0.35 | | 0.33 | 0.35 | |  |
| High γ Relative | 0.33 | 0.34 | 0.35 | | 0.38 | 0.26 | 0.39 | | 0.32 | 0.30 | |  |
| Slope 1-200 Hz | 0.82 | 0.87 | 0.87 | | 0.86 | 0.78 | 0.79 | | 0.83 | 0.79 | |  |
| Offset | 0.92 | 0.92 | 0.91 | | 0.93 | 0.92 | 0.89 | | 0.89 | 0.87 | |  |
| Slope 20-30 Hz | 0.64 | 0.70 | 0.69 | | 0.61 | 0.6 | 0.76 | | 0.72 | 0.61 | |  |
| Slope 20-40 Hz | 0.64 | 0.74 | 0.76 | | 0.69 | 0.62 | 0.73 | | 0.77 | 0.77 | |  |
| Slope 40-60 Hz | 0.6 | 0.71 | 0.76 | | 0.67 | 0.63 | 0.69 | | 0.71 | 0.76 | |  |
| Slope 30-45 Hz | 0.52 | 0.69 | 0.71 | | 0.66 | 0.56 | 0.75 | | 0.8 | 0.66 | |  |
| Slope 60-120 Hz | 0.55 | 0.68 | 0.7 | | 0.69 | 0.60 | 0.59 | | 0.67 | 0.77 | |  |
